# Precision Population Medicine in Primary Care: The Sanford Chip Experience

**DOI:** 10.3389/fgene.2021.626845

**Published:** 2021-03-12

**Authors:** Kurt D. Christensen, Megan Bell, Carrie L. B. Zawatsky, Lauren N. Galbraith, Robert C. Green, Allison M. Hutchinson, Leila Jamal, Jessica L. LeBlanc, Jennifer R. Leonhard, Michelle Moore, Lisa Mullineaux, Natasha Petry, Dylan M. Platt, Sherin Shaaban, April Schultz, Bethany D. Tucker, Joel Van Heukelom, Elizabeth Wheeler, Emilie S. Zoltick, Catherine Hajek, on behalf of the Imagenetics Metrics Team, Baye Jordan

**Affiliations:** Sanford Health: Jordan Baye, Megan Bell, Kristen Deberg, Benjamin Forred, Colette Free, Catherine Hajek, Joel Van Heukelom, Ashley Hopp, Allison Hutchinson, Ryne Lees, Jennifer Leonhard, Amanda Massmann, Michelle Moore, Amelia Mroch, Natasha Petry, Dylan Platt, Erin Royer, April Schultz, Murat Sincan, Bethany Tucker, and Elizabeth Wheeler. Harvard Pilgrim Health Care Institute: Kurt Christensen, Lauren Galbraith, Jessica LeBlanc, Ryan Walsh, and Emilie Zoltick. Brigham and Women’s Hospital: Robert Green, Charlene Preys, and Carrie Zawatsky. Mayo Clinic: Lisa Mullineaux. National Institutes of Health: Leila Jamal.; ^1^Center for Healthcare Research in Pediatrics, Department of Population Medicine, Harvard Pilgrim Health Care Institute, Boston, MA, United States; ^2^Department of Population Medicine, Harvard Medical School, Boston, MA, United States; ^3^Broad Institute of MIT and Harvard, Cambridge, MA, United States; ^4^Sanford Health Imagenetics, Sioux Falls, SD, United States; ^5^Department of Medicine, Brigham and Women’s Hospital, Boston, MA, United States; ^6^Ariadne Labs, Boston, MA, United States; ^7^Department of Medicine, Harvard Medical School, Boston, MA, United States; ^8^National Cancer Institute, Bethesda, MD, United States; ^9^Department of Bioethics, National Institutes of Health, Bethesda, MD, United States; ^10^Sanford Health Imagenetics, Bemidji, MN, United States; ^11^Mayo Clinic Genomics Laboratory, Rochester, MN, United States; ^12^Sanford Health Imagenetics, Fargo, ND, United States; ^13^Department of Pharmacy Practice, North Dakota State University, Fargo, ND, United States; ^14^Department of Pathology, University of Utah School of Medicine, Salt Lake City, UT, United States; ^15^ARUP Laboratories, Salt Lake City, UT, United States; ^16^Sanford School of Medicine, University of South Dakota, Sioux Falls, SD, United States

**Keywords:** pharmacogenomic testing, genetic counseling, decision support systems – clinical, genetic testing, primary health care

## Abstract

Genetic testing has the potential to revolutionize primary care, but few health systems have developed the infrastructure to support precision population medicine applications or attempted to evaluate its impact on patient and provider outcomes. In 2018, Sanford Health, the nation’s largest rural nonprofit health care system, began offering genetic testing to its primary care patients. To date, more than 11,000 patients have participated in the Sanford Chip Program, over 90% of whom have been identified with at least one informative pharmacogenomic variant, and about 1.5% of whom have been identified with a medically actionable predisposition for disease. This manuscript describes the rationale for offering the Sanford Chip, the programs and infrastructure implemented to support it, and evolving plans for research to evaluate its real-world impact.

## Introduction

Specialists are increasingly using genetic testing to accelerate diagnoses and improve treatment decisions after patients become sick. However, its true potential may be realized in unselected populations and preventive applications. Pharmacogenomic (PGx) information can be stored until a time of need, thereby avoiding potential life-threatening delays associated with reactive testing (O’Donnell et al., 2014; Relling and Evans, 2015; Weitzel et al., 2017). Patients could additionally be screened for inherited predispositions for conditions with effective preventive and early detection interventions that would otherwise remain unknown until disease onset (Amendola et al., 2015; Olfson et al., 2015; Kalia et al., 2017). Collaborative resources such as the Clinical Pharmacogenetics Implementation Consortium (CPIC), PharmGKB and ClinGen have emerged to develop and aggregate guidelines for genetic information, including its use in unselected populations (Klein et al., 2001; Relling and Klein, 2011; Rehm et al., 2015). Enthusiasm is growing for genomics as a component of precision population medicine, where disease treatment and prevention efforts for all patients are tailored to individuals’ genes, environments, and lifestyles (Goldenberg et al., 2013; Waisbren et al., 2016).

Many commentators anticipate a future in which such uses of genetic testing is standard of care for all patients, including primary care (Collins et al., 2003; Green et al., 2013b; McCarthy et al., 2013; Bell, 2017). Research shows that non-genetic specialist providers are receptive to integrating genetic testing into their practices when appropriately supported (Overby et al., 2014; Raghavan and Vassy, 2014), and that its results often satisfy the informational needs of patients (Roberts et al., 2018). Access to direct-to-consumer genetic testing (Allyse et al., 2018) and third-party services to re-interpret existing genomic data (Wang et al., 2018) is growing. Early projections also suggest that precision population medicine applications may be cost-effective (Bennette et al., 2015; Prince et al., 2017; Zhang et al., 2019). Yet, the challenges of integrating genetics into everyday patient care are daunting. The demands for specialized resources and trained personnel are considerable (Shuldiner et al., 2013; Hoskovec et al., 2018; Ginsburg et al., 2019), as most generalists, advanced practice providers, and nurses have had limited exposure to medical genetics and genomics during their training (Wolyniak et al., 2015; Campion et al., 2019). Few health systems have developed the infrastructure to store genomic data and facilitate clinical decision making (Kho et al., 2013; Williams et al., 2019). Moreover, limited evidence exists about the benefits and risks of population genetic testing, particularly within real-world clinical settings (Carey et al., 2016; Vassy et al., 2017; Manickam et al., 2018; Brothers et al., 2019; Stark et al., 2019). Further data about the impact of genetic testing applied in precision population medicine is critical to justify its implementation at a larger scale (Messner et al., 2017; Shaer et al., 2017).

In 2018, the Sanford Imagenetics Initiative began offering the Sanford Chip, a clinical laboratory-developed test that provides PGx information, as well as optional disease risk information, to adult patients across the Sanford Health system as part of primary care. This manuscript summarizes the environment and processes the Imagenetics Initiative developed to support and evaluate the Sanford Chip Program. The aim of this report is to offer an instructive example for implementing a precision population medicine program that emphasizes genomics (Rehm et al., 2015; National Cancer Institute, 2019; U.S. National Library of Medicine, 2020).

## The Sanford Health Imagenetics Initiative

**FIGURE 1:**
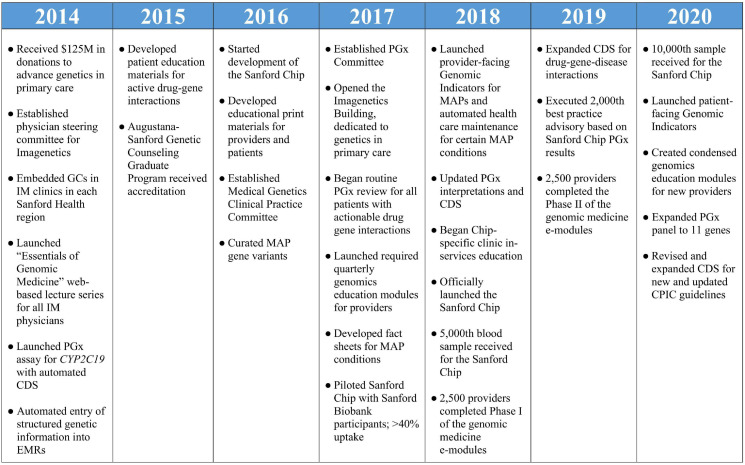
Milestones of the Imagenetics Initiative and Sanford Chip Program. GCs, genetic counselors; IM, internal medicine; PGx, pharmacogenomic; EMRs, electronic medical records; CDS, clinical decision support; MAP, medically actionable predispositions.

Sanford Health is the largest rural non-profit health system in the United States, serving more than 2 million patients through 46 medical centers, more than 200 clinics and 2,500 providers. Through strategic planning which included primary care providers (PCPs), genetics providers, pharmacists, and health system administrators initiated in 2012, Sanford Health identified that genomics should have an emerging role in the care of its patients. With a vision of the expansive role genetic testing could play in all aspects of medicine, Sanford Health developed an extensive plan for an “Imagenetics” (internal medicine and genetics) Initiative. With help from $125 million in external donations to fund this vision, Sanford Health launched the Imagenetics Initiative in 2014 with the goal of accelerating the implementation of genetic testing into primary care. Key milestones for the Imagenetics Initiative are summarized in Figure 1.

## Development of the Sanford Chip Program

### Testing Platform, Workforce, and Decision Support

**FIGURE 2:**
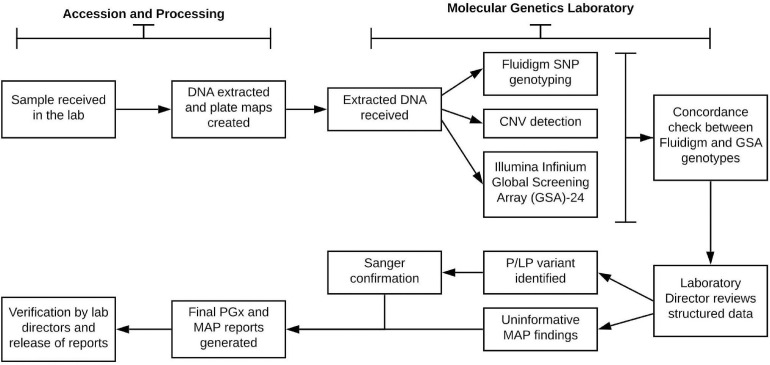
Laboratory process for pharmacogenomic testing and screening for medically actionable predispositions. SNP, single nucleotide polymorphism; P/LP, pathogenic or likely pathogenic; MAP, medically actionable predispositions.

A goal of the Imagenetics Initiative was to introduce a low-cost genomic test, the “Sanford Chip,” to enhance preventive care by providing PGx and disease risk information with the strongest evidence for actionability and the potential to improve patient outcomes. The Sanford Chip is offered to patients as an elective service. The laboratory processes for DNA testing, bioinformatic analyses, and reporting are summarized in Figure 2. For all participants enrolled in the Sanford Chip Program, preemptive PGx testing is conducted using the Fluidigm SNP Dynamic Array platform and TaqMan Assay for *CYP2D6* copy number assessment. To test for genetic risk factors associated with conditions with proven prevention options (“medically actionable predispositions,” or “MAPs”), the program used Illumina’s Infinium Global Screening Array-24 (GSA). The array captured the entire GWAS catalog as of May 2016 (Buniello et al., 2019), and was customized to include additional markers associated with cardiovascular, oncologic, and neurologic traits for future applications. PGx results are compared to the GSA for concordance. MAPs are confirmed using Sanger sequencing. Testing is performed at the CLIA-certified and CAP-accredited Molecular Genetics Laboratory of Sanford Imagenetics (Sioux Falls, SD). Certified molecular geneticists review quality control metrics for each sample, confirm whether patients have consented for review of MAP variants, and oversee variant interpretation and reporting as applicable. The genes currently examined by the Sanford Chip and their associations with medications and disease are summarized in Supplementary Tables 1,2.

The Imagenetics Initiative developed the technical and clinical expertise to manage the anticipated increased demand for genetic testing system-wide with the roll-out of the Sanford Chip. Sanford Health hired and embedded genetic counselors in primary care clinics, starting with internal medicine clinics in its major markets (Sioux Falls, SD, Fargo, ND, Bismarck, ND, Bemidji, MN) to help ensure patients and providers had the resources to make informed choices about testing and responding to results. This approach has since been expanded to embed genetic counselors in additional primary care clinics, including family medicine and obstetrics and gynecology. To address the shortage of genetic counselors nationally (Hoskovec et al., 2018), Sanford Health partnered with Augustana University to establish the Augustana-Sanford Master of Science in Genetic Counseling Program. This full-time training program, accredited in 2015, has helped develop a pool of qualified professionals who are familiar with the Imagenetics Initiative. The Imagenetics Initiative also leveraged and continues to lean on Sanford Health’s telegenetics expertise to facilitate access for patients who may live far from urban centers. These capabilities are particularly important, given the rural nature of the Sanford Health System.

Clinical decision support (CDS) was developed for Sanford Chip findings using provider and patient Genomic Indicators. Genomic Indicators are functions specific to the EPIC electronic medical records (EMRs) system that facilitate the use of genetic information, including storage of PGx information and findings about MAPs (Caraballo et al., 2020). Indicators appear in the patient summary section of the EMR as well as within the patient’s MyChart record.

### Pharmacogenomic Testing

Details about the development of Sanford Health’s PGx program have been published previously (Petry et al., 2019), and points relevant to the Sanford Chip Program are briefly re-stated here. The Imagenetics Initiative formed a system-wide interdisciplinary Pharmacogenomics Committee (PGx Committee) to oversee the PGx program, including prioritization of drug-gene relationships and development of guidance for using PGx information to inform drug selection and dosing decisions. The PGx committee also established a process for developing CDS to ensure that PGx information would be integrated into patients’ EMRs, and that the information would be available at the time of prescribing.

Based on PGx information, automated CDS for 63 drug-gene interactions and 3 drug-gene-disease interactions informs providers that patients may benefit from alterations to medication orders. Examples of the CDS developed for voriconazole orders for patients with *CYP2C19* rapid metabolizer genotypes is presented in the Supplementary Image 1. Automated CDS alerts were also developed for drug-gene-disease interactions that may be informed by genetic screening. For instance, CDS notifies providers to avoid medications that may prolong the QT interval in patients with long QT syndrome (Priori et al., 2013; Al-Khatib et al., 2018). CDS is evaluated annually by PGx pharmacists with specialty-specific clinicians and updated to match CPIC^®^ or other consensus guidelines. Patient-facing Genomic Indicators are also sent to MyChart and include a short summary of the PGx findings and uninformative or disease risk MAP findings in plain language, and encourages patients to contact Imagenetics specialists for more information. Notably, CDS was considered for instances when medication orders were consistent with CPIC^®^ guidelines, but ultimately omitted to avoid “alert fatigue” (Shojania et al., 2010; Slight et al., 2013; Bryant et al., 2014; Hinderer et al., 2017; Tolley et al., 2018).

### Screening for Medically Actionable Predispositions

The Imagenetics Initiative simultaneously developed approaches to screen patients for MAPs. A Medical Genetics Clinical Practice Committee (CPC) was established to include a group of medical professionals including physicians, genetic counselors, and pharmacists to develop practice guidelines for the Sanford Health system. The Medical Genetics CPC opted to screen for variants in genes recommended by the American College of Medical Genetics and Genomics (ACMG) for secondary findings disclosure (Green et al., 2013a; Kalia et al., 2017). The committee recognized that the ACMG list of genes was not intended to guide or endorse population genetic screening (ACMG Board of Directors, 2019). Nevertheless, it felt that the deliberations about the actionability of these genes (Green et al., 2012) and development of resources to inform clinical responses for unselected populations (Clinical Genome Resource, 2020) represented a consensus about genes for which disclosure would have the best likelihood of leading to clinical benefits.

The Medical Genetics CPC has considered expanding the gene list to screen for additional genes and conditions. The ACMG list of genes was intended to be a “minimal list” for secondary findings disclosure (Green et al., 2013a), and programs that screen biobank participants for MAPs, such as the Geisinger MyCode initiative, currently screen more genes associated with conditions (Carey et al., 2016). Given considerable concern about population genetic screening (Prince et al., 2017; ACMG Board of Directors, 2019; Brothers et al., 2019), Sanford Health has opted for a conservative approach. However, the Medical Genetics CPC reviews the list twice annually to discuss whether changes should be made, including adding or removing conditions and genes.

The laboratory identifies and reports variants on the Sanford Chip that it classifies as pathogenic or likely pathogenic (Richards et al., 2015). Genomic Indicators for MAP findings are automatically integrated into the EMRs of Sanford Chip recipients. Their use automatically modifies recommended surveillance according to best practice guidelines.

## Preparing Providers and Patients for the Sanford Chip Program

A physician steering committee with representatives from internal medicine, family medicine, pediatrics, and obstetrics designed an educational program for both health care providers and for administrators of the Sanford Health System. Prior to the launch of the Sanford Chip test, a combination of live and recorded lectures was made available to all providers. From 2017 to 2019, more than 2,500 Sanford physicians and advanced practice providers were required to complete quarterly continuing medical education focused on genetic medicine, with an emphasis on preparing providers to be able to respond to Sanford Chip requests and results. The modules were also offered to all Sanford Health administrators. Details about the content and impact of this educational program are the focus of ongoing research and forthcoming publications. Since the launch of the Sanford Chip, ongoing educational efforts have included in-services, recorded modules and internal and external websites which host repositories for educational resources for health care teams and patients.

## The Patient Experience

**FIGURE 3:**
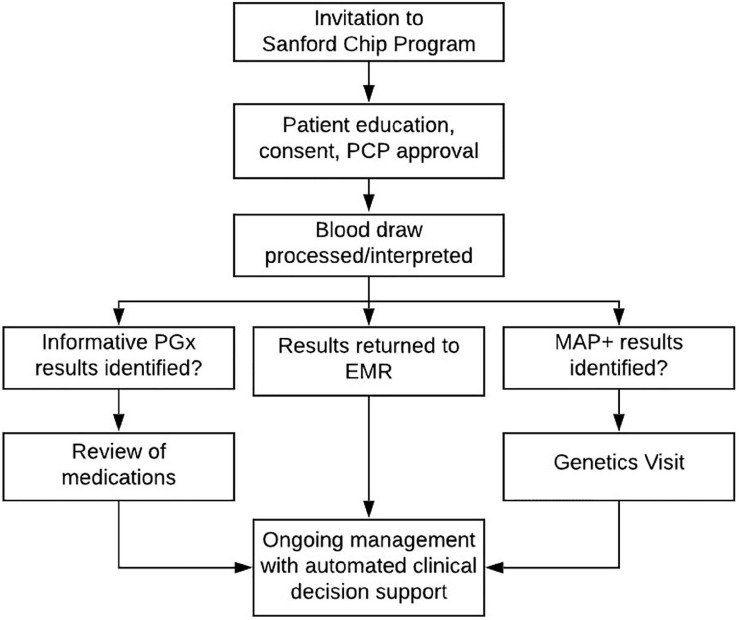
Process for testing, return of results, and ongoing patient management. EMR, electronic medical record; PGx, pharmacogenomic results; MAP, findings about medically actionable predispositions; CDS, clinical decision support.

The Imagenetics Initiative began to offer the Sanford Chip to patients in 2018. An overview of the current and ongoing process for inviting, testing, and reporting Sanford Chip results is summarized in Figure 3. Adult patients are eligible to receive the Sanford Chip if they receive primary care (internal medicine, family medicine, or obstetrics/gynecology) within the Sanford Health System and have a Sanford MyChart account through which they can receive communications and access laboratory findings. The most common way eligible patients receive the Sanford Chip is by responding to invitations sent through MyChart. Links that are embedded in the invitations direct patients to a secure web-based platform where they provide clinical consent for testing. During the process, there is an option to request contact with a laboratory genetic counselor via the medium of their choice (phone, secure MyChart message). Patients who provide clinical consent report their personal and family histories of disease on questionnaires. Patients’ PCPs approve the request to receive the Sanford Chip. In less than 20 cases the patient’s PCP did not approve, and the order was forwarded to a dedicated physician in each region for further consideration and approval, if appropriate. Patients then have a blood draw at any Sanford laboratory for genetic screening. To date, more than 11,000 patients have participated in the Sanford Chip Program, 62% of whom are female and 38% of whom are male. Participating women and men are 50 and 58 years old on average, respectively, and have an average of 9.4 and 8.9 medications on their medication lists, respectively. Patients who participate in the Sanford Chip Program are slightly older than Sanford Health patients overall (average age of women: 48; average age of men: 52) and taking more medications (average medications for men: 7.3; average medications for women: 7.8). Over 90% of participants have been identified with at least one informative pharmacogenomic variant (Supplementary Table 3). Approximately 1.5% of participants who agreed to screening for MAPs have been identified with an autosomal dominant disease predisposition, as summarized in Table 1. Another 1.6% and 0.8% of individuals have been identified with variants in *MUTYH* and *ATP7B*, respectively. These individuals received genetic counseling regarding their carrier status and were offered full sequencing to confirm whether they are homozygous or compound heterozygous for pathogenic variants. The process for inviting, consenting, and ultimately communicating results is periodically reviewed by Sanford legal, compliance and privacy offices.

**TABLE 1:** Frequency of pathogenic and likely pathogenic variants in genes screened for medically actionable predispositions (MAPs).

**Condition**	**Gene**	***n* (%)**
Cardiomyopathy/arrhythmogenic right	*MYBPC3*	26 (0.22)
ventricular cardiomyopathy	*TNNT2*	12 (0.10)
	*MYH7*	5 (0.04)
	*DSC2*	1 (0.01)
	*DSG2*	1 (0.01)
	*DSP*	1 (0.01)
	*GLA*	1 (0.01)
	*LMNA*	1 (0.01)
	*MYL2*	2 (0.02)
	*PKP2*	2 (0.02)
	*TNNI3*	2 (0.02)
Hereditary breast and other cancers	*BRCA1*	23 (0.19)
	*BRCA2*	17 (0.14)
	*TP53*	2 (0.02)
Familial hypercholesterolemia	*APOB*	16 (0.13)
	*LDLR*	10 (0.08)
Hereditary colon cancer	*MSH6*	7 (0.06)
	*PMS2*	6 (0.05)
	*MLH1*	1 (0.01)
	*MSH2*	1 (0.01)
Long QT syndrome	*KCNQ1*	11 (0.09)
	*SCN5A*	8 (0.07)
	*KCNH2*	2 (0.02)
Malignant hyperthermia	*RYR1*	14 (0.12)
	*CACNA1S*	1 (0.01)
Hereditary paraganglioma	*SDHB*	1 (0.01)
	*SDHC*	1 (0.01)
Ehlers–Danlos syndrome	*COL3A1*	1 (0.01)
Thoracic aortic aneurysms	*TGFBR2*	1 (0.01)

*Results represent findings in the first 11,874 Sanford Chip recipients who agreed to screening.*

### Return of Sanford Chip Results

Sanford Chip results are automatically placed into the EMRs via a Health Level Seven interface to facilitate CDS. Simultaneously, automated alerts inform laboratory genetic counselors that results are ready for provider and patient notification. Final reports are sent to ordering providers’ queues for review. Upon release of structured data into the EMR, a message is sent to an in-basket leading to a complete retrospective review of drug-gene variants by a clinical pharmacist. Clinical pharmacists review every patient’s PGx results, medication lists, and clinical profiles, and provide recommendations for alterations, if needed, to PCPs through a clinical note within the patient’s medical record. PCPs have discretion about whether to discuss PGx results with the patient or to change existing medications. Future prescribing of medications affected by a patient’s PGx results is supported by programmed CDS.

When MAPs are identified, laboratory genetic counselors call the ordering providers’ offices and discuss the findings with the providers or their staff. The laboratory genetic counselors then contact patients to review the results and offer clinical appointments with genetic specialists to discuss the findings more thoroughly. Reports are released to patients via their MyChart portal no later than 14 days after reports are drafted. At subsequent clinical appointments with a specialist, comprehensive family histories are collected and reviewed. Genetic counselors discuss the variants and the associated conditions as well as any health management or screening recommendations, along with implications for other family members. At the end of the patient visit, the genetic counselor reviews and initiates additional referrals and follow-up genetic testing as appropriate.

Patients who are not identified with reportable variants in the MAP gene list are informed via MyChart that they have “uninformative results” regarding disease risks. The emphasis on results being “uninformative” rather than “negative” is conventional for genetic testing in the absence of a known familial variant, to minimize the risk that patients will interpret a lack of findings to mean that they have no pathogenic variants for a genetic disorder (Uhlmann et al., 2009). These risks may be even greater for array-based genetic screening approaches that screen for pre-specified list of pathogenic variants, as is currently used for the Sanford Chip Program, given that many causal variants are unique to individuals or families (Alfares et al., 2018). Ordering providers have the option to release the uninformative results, with or without standardized verbiage that emphasizes these limitations of the Sanford Chip to identify disease risks, to the patient portal or allow the system to release the results after 14 days. Follow-up of uninformative results is done at the ordering providers’ discretion.

## Evaluating Outcomes: Imagenetics Metrics

Given the robust genomics infrastructure that was established, the Imagenetics Initiative and Sanford Chip Program also provide a real-world setting to generate evidence about its impact on providers and patients (Khoury et al., 2018; Murray et al., 2019). In 2019, Sanford Health began a collaboration with investigators at the Harvard Medical School to launch the Imagenetics Medical/Economic Impact and Reactions to the Sanford Chip Study (METRICS). This research collaboration has focused its initial work on four key aspects of this precision population medicine program: provider preparedness, PGx testing, medically actionable findings, and uninformative MAP findings.

## Discussion

With the Sanford Chip, the Imagenetics Initiative implemented one of the first genetics-focused precision population medicine programs that is fully integrated into a health system. Nearly five years prior to offering the Sanford Chip, Sanford Health’s Imagenetics Initiative began developing the plans and infrastructure to support genetic testing in primary care settings. This work included creating the appropriate governance structure, recruiting appropriate personnel, expanding the laboratory and clinical capacity, creating decision support, and ensuring providers were educated and supported. The approach we have summarized here provides a real-world example for implementing genetic testing to inform preventive care in the future, and illuminates the complex planning involved in launching such an enterprise.

The infrastructure that the Imagenetics Initiative created to prepare for the Sanford Chip required a significant financial and strategic commitment from Sanford Health. The support provided to the Sanford Chip Program allowed the Imagenetics Initiative to establish a laboratory and informatics pipeline appropriate for genetic testing at a large scale and create education programs and CDS to support health care providers without specialized training in genetics. Additional investments support retrospective review of pharmacologic information of all Sanford Chip recipients and robust follow-up with patients who are identified with MAPs. The prices charged to payers (primarily patients) to receive the Sanford Chip, are unlikely to cover the costs to develop the program, but current projections about preemptive pharmacogenomic testing and genetic screening show encouraging evidence about their cost effectiveness from a health sector and societal perspective (Plumpton et al., 2019; Zhang et al., 2019; Dong et al., 2020). Nevertheless, health systems that expect precision population medicine programs to be revenue-neutral from the start are likely to find it challenging to ensure providers are maximizing the potential benefits of genetic information for patients (Dzau et al., 2016; Gaff et al., 2017; Levy et al., 2019).

The Sanford experience also provides an instructive example for training and supporting health care providers for precision population medicine system-wide. The Imagenetics Initiative developed ongoing educational curricula tailored to the needs of both generalist and specialist providers. It also developed automated CDS that informs providers about how the results from PGx and genetic screening can inform medical decision making at the time the information is needed. Two aspects of Sanford’s provider education approach merit particular emphasis. First, genetics education over a 2-year period was mandatory for all physicians and advanced practice providers. While other health systems have implemented or capitalized on large-scale provider education efforts for genetics (Murray, 2014; Rubanovich et al., 2018; Crellin et al., 2019), few have had the institutional commitment to require them over long periods of time. Second, the Sanford-Augustana genetic counseling training program not only helps address a national shortage of genetic counselors, but its graduates who take positions at Sanford Health are already familiar with the Sanford patient population and the Sanford Chip.

The challenges of implementing genetics-focused precision population medicine programs are exacerbated by a dearth of guidelines for managing healthy patients with genetic predispositions who have no personal or family history of disease. The current landscape may encourage management practices that may overtreat patients (Woolever, 2008; Caulfield et al., 2013; Diamandis, 2015). Uncertainties about the downstream impact of genetic screening have made payers reluctant to cover genetic screening (Phillips et al., 2014). In order for the field to realize the benefits of genetic screening, it will be important to establish clinical guidelines which facilitate consistent management of asymptomatic patients.

The inclusion of a dedicated research component (METRICS) is another notable aspect of Sanford’s precision population medicine program. While the evidence base for genomic medicine from clinical research is growing rapidly, there is a need to complement these efforts with real-world evidence that follows clinical implementation (Sherman et al., 2016; Berger et al., 2017). METRICS will explore the impact of integrating genetic testing into general clinical practice in an environment that has successfully developed the infrastructure and processes to support it. Moreover, the research will collect patient-reported outcomes that are sometimes omitted from evaluations of precision medicine innovations (Glasgow et al., 2018).

The current approach used to implement the Sanford Chip Program has limitations. PGx testing and genetic screening of healthy patients is voluntary and not standard of care (U.S. Food and Drug Administration, 2018; ACMG Board of Directors, 2019). Experiences about and data from the Sanford Chip Program will help address knowledge gaps about the impact of genomic testing in rural populations (Mapes et al., 2020). However, the areas served by Sanford Health are primarily white, and the work pursued by Sanford Health may provide limited insight to address well-recognized issues about health disparities and underrepresentation of marginalized communities that genomic testing may exacerbate (Landry and Rehm, 2018; Kim and Sarkar, 2019; Martin et al., 2019; Roberts et al., 2019). The array used for genetic screening is less sensitive for identifying pathogenic genetic variants than approaches such as exome or genome sequencing, as noted previously, necessitating a strong educational program to explain the limitations. As the applications for genomics in medicine grow, so should educational content in medical training to address such limitations.

Despite its limitations, the decision to use array technology to launch the Sanford Chip Program provided a lower cost platform for which the infrastructure for supporting a precision population genomic medicine approach could be implemented. The infrastructure that Sanford Health developed positions it to shift to more testing approaches such as exome or genome sequencing in the future. Moreover, the Sanford Chip Program provides the ability for patients to obtain genomic testing in a medical setting that they might otherwise seek from direct-to-consumer options that may omit the expertise to guide interpretation and ongoing management of genetic testing results. The health system chose to pursue a clinical approach to address this patient demand and provide genetic information in a medically responsible manner.

Future versions of the Sanford Chip may include polygenic risk predictions for common, complex conditions, additional PGx information, more comprehensive coverage of MAP genes, or other disease-gene relationships deemed appropriate for clinical return in healthy populations. Looking ahead, we envision an environment where the inclusion of genomics in the care of patients is standard and contributes to more precise risk assessment and management. The Sanford Chip Program is an important step in achieving this vision and will inform the future of genomic medicine not only in the Sanford Health system, but at health systems worldwide.

## Members of The Imagenetics Metrics Team

Sanford Health: Jordan Baye, Megan Bell, Kristen Deberg, Benjamin Forred, Colette Free, Catherine Hajek, Joel Van Heukelom, Ashley Hopp, Allison Hutchinson, Ryne Lees, Jennifer Leonhard, Amanda Massmann, Michelle Moore, Amelia Mroch, Natasha Petry, Dylan Platt, Erin Royer, April Schultz, Murat Sincan, Bethany Tucker, and Elizabeth Wheeler. Harvard Pilgrim Health Care Institute: Kurt Christensen, Lauren Galbraith, Jessica LeBlanc, Ryan Walsh, and Emilie Zoltick. Brigham and Women’s Hospital: Robert Green, Charlene Preys, and Carrie Zawatsky. Mayo Clinic: Lisa Mullineaux. National Institutes of Health: Leila Jamal.

## Data Availability Statement

The raw data supporting the conclusions of this article will be made available by the authors, without undue reservation.

## Ethics Statement

The studies involving human participants were reviewed and approved by the Sanford Health Institutional Review Board, including a waiver of informed consent to analyze de-identified, aggregated data.

## Author Contributions

MB, AH, JRL, MM, LM, NP, DP, SS, AS, BT, JV, EW, and CH contributed to the conception and design of the Imagenetics Initiative and have done clinical and educational work at Sanford Health. KC, CZ, LG, RCG, JLL, LJ, and EZ helped guide analysis of these clinical and educational interventions. The provider preparedness efforts have been led by AH, DP, and CH, with team members JLL, LJ, AS, EW, NP, EZ, LG, DP, KC, and CH contributing to the design of the study and data analysis. Clinical and research efforts regarding medically actionable predispositions have been led by LM, BT, and MM, with team members EZ, MM, BT, KC, LG, CH, JV, CZ, AS, and JRL contributing to the design of the study. Clinical and research efforts regarding pharmacogenomic results have been led by AS, with team members EZ, JV, KC, LG, CH, and NP contributing to the design of the study. Clinical and research efforts regarding uninformative findings have been led by MB, with team members DP, JLL, EZ, KC, LG, CZ, BT, JRL, and CH contributing to the design of the study. All authors revised the manuscript, and approved the final submitted version.

## Conflict of Interest

RCG has received compensation for advising the following companies: AIA, Grail, Humanity, Kneed Media, Plumcare, UnitedHealth, Verily, VibrentHealth, Wamberg; and is co-founder of Genome Medical, Inc., a technology and services company providing genetics expertise to patients, providers, employers and care systems. The remaining authors declare that the research was conducted in the absence of any commercial or financial relationships that could be construed as a potential conflict of interest.

## Supplementary Material

Supplementary Image 1 Examples of clinical decision support alerts for patients with *CYP2C19* rapid metabolizer status. The top example is the alert that activates for an adult patient, while the bottom example is the alert that activates for a pediatric patient.

Supplementary Table 1 Summary of pharmacogenomic variants and medically actionable predispositions that are targeted by the Sanford Chip.

Supplementary Table 2 Summary of medically actionable predispositions that are targeted by the Sanford Chip.

Supplementary Table 3 Frequency of phenotypes/genotypes in pharmacogenomic genes. Results represent findings from the first 10,723 patients who enrolled in the Sanford Chip program. Counts vary by gene depending on the genes included on the Sanford Chip at the time patients received testing. Some genes from Supplementary Table 1 are omitted because they were only recently added to the Sanford Chip.
