# Supplementary material for: Precision Population Medicine in Primary Care: The Sanford Chip Experience
Source: Front Genet. 2021 Mar 12;12:626845. doi: 10.3389/fgene.2021.626845 (PMC7994529; doi:10.3389/fgene.2021.626845)

Image S1. Examples of clinical decision support alerts for patients with *CYP2C19* rapid metabolizer status. The top example is the alert that activates for an adult patient, while the bottom example is the alert that activates for a pediatric patient.

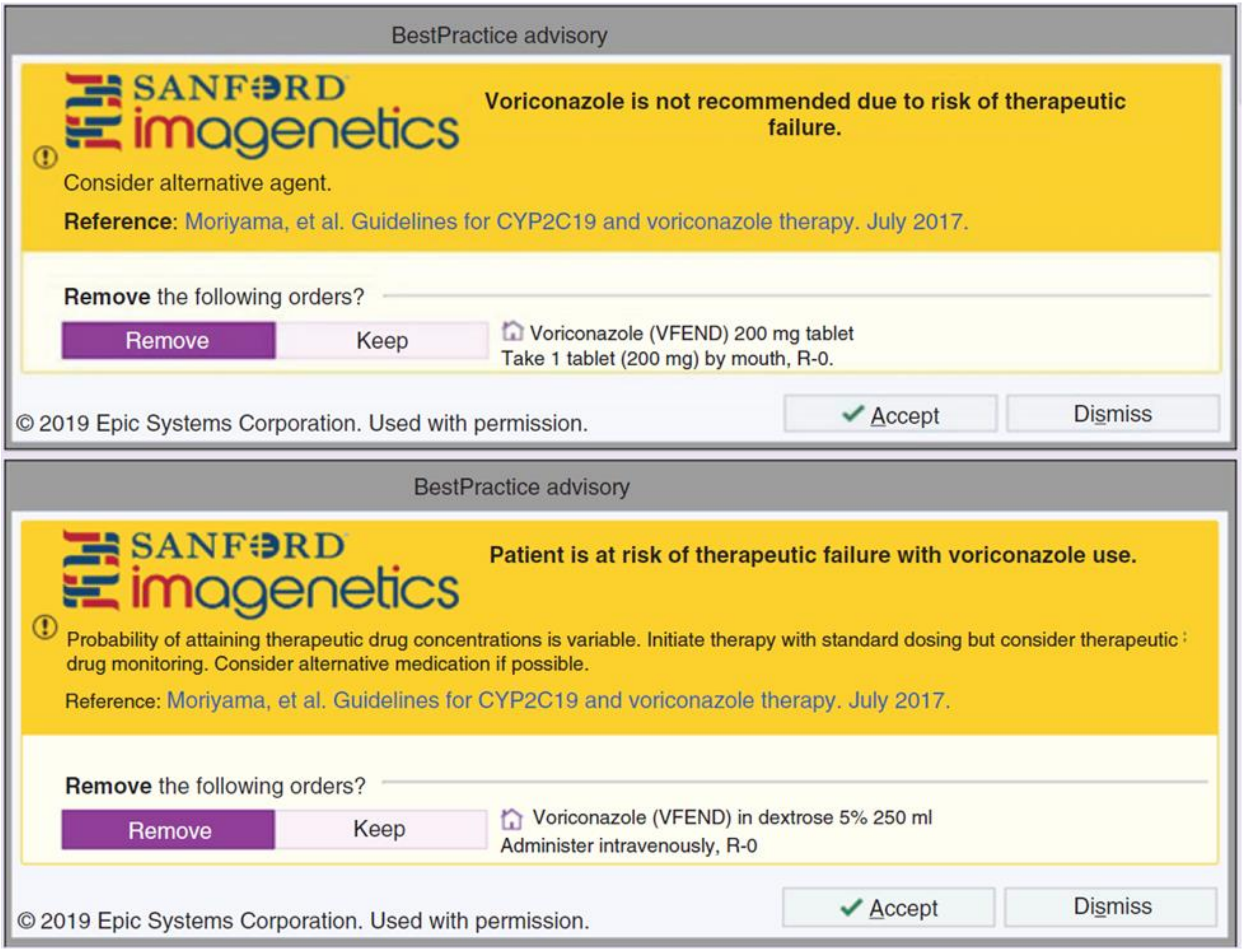

Supplement: Supplementary Image 1 — Examples of clinical decision support alerts for patients with CYP2C19 rapid metabolizer status. The top example is the alert that activates for an adult patient, while the bottom example is the alert that activates for a pediatric patient. [file Image_1.pdf]
