# Supplementary material for: Precision Population Medicine in Primary Care: The Sanford Chip Experience
Source: Front Genet. 2021 Mar 12;12:626845. doi: 10.3389/fgene.2021.626845 (PMC7994529; doi:10.3389/fgene.2021.626845)
Supplement: Supplementary Table 1 — Summary of pharmacogenomic variants and medically actionable predispositions that are targeted by the Sanford Chip. [file Table_1.docx]

Table S1. Summary of pharmacogenomic variants that are targeted by the Sanford Chip.

| **Pharmacogenomic Testing** | |  |
| --- | --- | --- |
| **Gene(s)** | **Medications** | **Clinical Pharmacogenetics Implementation Consortium Guidelines** |
|  |  |  |
| **Anticonvulsants** |  |  |
| *CYP2C9* | · Fosphenytoin | Karnes et al., 2021 |
|  | · Phenytoin |  |
|  |  |  |
| ***Antidepressants*** |  |  |
| *CYP2D6 & CYP2C19* | · Amitriptyline | Hicks et al., 2017;Caudle et al., 2020;Clinical Pharmacogenetics Implementation Consortium, 2021c |
|  | · Citalopram |  |
|  | · Clomipramine |  |
|  | · Desipramine |  |
|  | · Doxepin |  |
|  | · Escitalopram |  |
|  | · Fluoxetine |  |
|  | · Fluvoxamine |  |
|  | · Imipramine |  |
|  | · Nortriptyline |  |
|  | · Paroxetine |  |
|  | · Sertraline |  |
|  | · Trimipramine |  |
|  |  |  |
| ***Antifungal Medications*** | |  |
| *CYP2C19* | · Voriconazole | Moriyama et al., 2017 |
|  |  |  |
| ***Antinausea Medications*** | |  |
| *CYP2D6* | · Ondansetron | Bell et al., 2017;Caudle et al., 2020 |
|  |  |  |
| ***Blood Thinners*** |  |  |
| *CYP2C19* | · Clopidogrel | Scott et al., 2013 |
| *CYP2C9, VKORC1, CYP4F2, & CYP2C* gene cluster | · Warfarin | Johnson et al., 2017 |
|  |  |  |
| ***Chemotherapeutic Agents*** | |  |
| *DPYD* | · Capecitabine | Amstutz et al., 2018;Clinical Pharmacogenetics Implementation Consortium, 2021a |
|  | · Fluorouracil |  |
| *TPMT* | · Mercaptopurine | Relling et al., 2019;Clinical Pharmacogenetics Implementation Consortium, 2021b |
|  | · Thioguanine |  |
|  |  |  |

| ***Cholesterol Medications*** | |  |
| --- | --- | --- |
| *SLCO1B1* | · Simvastatin | Ramsey et al., 2014 |
|  |  |  |
| ***Immunosuppressive Agents*** | |  |
| *CYP3A5* | · Tacrolimus | Birdwell et al., 2015 |
| *IFNL3* | · Interferon alpha | Muir et al., 2014 |
| *TPMT* | · Azathioprine | Relling et al., 2019;Clinical Pharmacogenetics Implementation Consortium, 2021b |
|  |  |  |
| ***Pain Medications*** | |  |
| *CYP2D6* | · Codeine | Crews et al., 2021 |
|  | · Tramadol |  |
|  |  |  |
| ***Protein Pump Inhibiters*** | |  |
| *CYP2C19* | · Dexlansoprazole  · Lansoprazole  · Omeprazole  · Pantoprazole | Lima et al., In press |
|  |  |  |
|  |  |  |
| ***Nonsteroidal Anti-inflammatory Drugs*** | | |
| *CYP2C9* | · Celecoxib | Theken et al., 2020 |
|  | · Flurbiprofen |  |
|  | · Ibuprofen |  |
|  | · Meloxicam |  |
|  | · Piroxicam |  |

**REFERENCES, TABLE S1**

Amstutz, U., Henricks, L.M., Offer, S.M., Barbarino, J., Schellens, J.H.M., Swen, J.J., et al. (2018). Clinical Pharmacogenetics Implementation Consortium (CPIC) guideline for dihydropyrimidine dehydrogenase genotype and fluoropyrimidine dosing: 2017 update. *Clin Pharmacol Ther.* 103**,** 210-216. doi: 10.1002/cpt.911.

Bell, G.C., Caudle, K.E., Whirl-Carrillo, M., Gordon, R.J., Hikino, K., Prows, C.A., et al. (2017). Clinical Pharmacogenetics Implementation Consortium (CPIC) guideline for CYP2D6 genotype and use of ondansetron and tropisetron. *Clin Pharmacol Ther.* 102**,** 213-218. doi: 10.1002/cpt.598.

Birdwell, K.A., Decker, B., Barbarino, J.M., Peterson, J.F., Stein, C.M., Sadee, W., et al. (2015). Clinical Pharmacogenetics Implementation Consortium (CPIC) guidelines for CYP3A5 genotype and tacrolimus dosing. *Clin Pharmacol Ther.* 98**,** 19-24. doi: 10.1002/cpt.113.

Caudle, K.E., Sangkuhl, K., Whirl-Carrillo, M., Swen, J.J., Haidar, C.E., Klein, T.E., et al. (2020). Standardizing CYP2D6 genotype to phenotype translation: consensus recommendations from the Clinical Pharmacogenetics Implementation Consortium and Dutch Pharmacogenetics Working Group. *Clin Transl Sci.* 13**,** 116-124. doi: 10.1111/cts.12692.

Clinical Pharmacogenetics Implementation Consortium (2021a). *CPIC® Guideline for Fluoropyrimidines and DPYD* [Online]. Available: <https://cpicpgx.org/guidelines/guideline-for-fluoropyrimidines-and-dpyd/> [Accessed Feb 8 2021].

Clinical Pharmacogenetics Implementation Consortium (2021b). *CPIC® Guideline for Thiopurines and TPMT and NUDT15* [Online]. Available: <https://cpicpgx.org/guidelines/guideline-for-thiopurines-and-tpmt/> [Accessed Feb 8 2021].

Clinical Pharmacogenetics Implementation Consortium (2021c). *CPIC® Guideline for Tricyclic Antidepressants and CYP2D6 and CYP2C19* [Online]. Available: <https://cpicpgx.org/guidelines/guideline-for-tricyclic-antidepressants-and-cyp2d6-and-cyp2c19/> [Accessed Feb 8 2021].

Crews, K.R., Monte, A.A., Huddart, R., Caudle, K.E., Kharasch, E.D., Gaedigk, A., et al. (2021). Clinical Pharmacogenetics Implementation Consortium (CPIC) guideline for CYP2D6, OPRM1, and COMT genotype and select opioid therapy. *Clin Pharmacol Ther.* doi: 10.1002/cpt.2149.

Hicks, J.K., Sangkuhl, K., Swen, J.J., Ellingrod, V.L., Müller, D.J., Shimoda, K., et al. (2017). Clinical pharmacogenetics implementation consortium guideline (CPIC) for CYP2D6 and CYP2C19 genotypes and dosing of tricyclic antidepressants: 2016 update. *Clin Pharmacol Ther.* 102**,** 37-44. doi: 10.1002/cpt.597.

Johnson, J.A., Caudle, K.E., Gong, L., Whirl-Carrillo, M., Stein, C.M., Scott, S.A., et al. (2017). Clinical Pharmacogenetics Implementation Consortium (CPIC) guideline for pharmacogenetics-guided warfarin dosing: 2017 update. *Clin Pharmacol Ther.* 102**,** 397-404. doi: 10.1002/cpt.668.

Karnes, J.H., Rettie, A.E., Somogyi, A.A., Huddart, R., Fohner, A.E., Formea, C.M., et al. (2021). Clinical Pharmacogenetics Implementation Consortium (CPIC) guideline for CYP2C9 and HLA-B genotypes and phenytoin dosing: 2020 update. *Clin Pharmacol Ther.* 109**,** 302-309. doi: 10.1002/cpt.2008.

Lima, J.J., Thomas, C.D., Barbarino, J., Desta, Z., Van Driest, S.L., El Rouby, N., et al. (In press). Clinical Pharmacogenetics Implementation Consortium (CPIC) guideline for CYP2C19 and proton pump inhibitor dosing. *Clin Pharmacol Ther.* doi: 10.1002/cpt.2015.

Moriyama, B., Obeng, A.O., Barbarino, J., Penzak, S.R., Henning, S.A., Scott, S.A., et al. (2017). Clinical Pharmacogenetics Implementation Consortium (CPIC) guidelines for CYP2C19 and voriconazole therapy. *Clin Pharmacol Ther.* 102**,** 45-51. doi: 10.1002/cpt.583.

Muir, A.J., Gong, L., Johnson, S.G., Lee, M.T., Williams, M.S., Klein, T.E., et al. (2014). Clinical Pharmacogenetics Implementation Consortium (CPIC) guidelines for IFNL3 (IL28B) genotype and PEG interferon-α-based regimens. *Clin Pharmacol Ther.* 95**,** 141-146. doi: 10.1038/clpt.2013.203.

Ramsey, L.B., Johnson, S.G., Caudle, K.E., Haidar, C.E., Voora, D., Wilke, R.A., et al. (2014). The clinical pharmacogenetics implementation consortium guideline for SLCO1B1 and simvastatin-induced myopathy: 2014 update. *Clin Pharmacol Ther.* 96**,** 423-428. doi: 10.1038/clpt.2014.125.

Relling, M.V., Schwab, M., Whirl-Carrillo, M., Suarez-Kurtz, G., Pui, C.H., Stein, C.M., et al. (2019). Clinical Pharmacogenetics Implementation Consortium guideline for thiopurine dosing based on TPMT and NUDT15 genotypes: 2018 update. *Clin Pharmacol Ther.* 105**,** 1095-1105. doi: 10.1002/cpt.1304.

Scott, S.A., Sangkuhl, K., Stein, C.M., Hulot, J.S., Mega, J.L., Roden, D.M., et al. (2013). Clinical Pharmacogenetics Implementation Consortium guidelines for CYP2C19 genotype and clopidogrel therapy: 2013 update. *Clin Pharmacol Ther.* 94**,** 317-323. doi: 10.1038/clpt.2013.105.

Theken, K.N., Lee, C.R., Gong, L., Caudle, K.E., Formea, C.M., Gaedigk, A., et al. (2020). Clinical Pharmacogenetics Implementation Consortium guideline (CPIC) for CYP2C9 and nonsteroidal anti-inflammatory drugs. *Clin Pharmacol Ther.* 108**,** 191-200. doi: 10.1002/cpt.1830.
