# Supplementary material for: Precision Population Medicine in Primary Care: The Sanford Chip Experience
Source: Front Genet. 2021 Mar 12;12:626845. doi: 10.3389/fgene.2021.626845 (PMC7994529; doi:10.3389/fgene.2021.626845)
Supplement: Supplementary Table 2 — Summary of medically actionable predispositions that are targeted by the Sanford Chip. [file Table_2.docx]

Table S2. Summary of medically actionable predispositions that are targeted by the Sanford Chip.

| **Screening for Medically Actionable Predispositions** | | |  | |
| --- | --- | --- | --- | --- |
| **Gene(s)** | **Conditions** | | **ClinGen Actionability Summary Report** | |
|  |  | |  | |
| ***Cancer Predispositions*** |  | |  | |
| *APC* | Familial adenomatous polyposis | | ClinGen Clinical Genome Resource, 2021c | |
| *BMPR1A, SMAD4* | Juvenile polyposis | | ClinGen Clinical Genome Resource, 2021x | |
| *BRCA1, BRCA2* | Hereditary breast and ovarian cancer | | ClinGen Clinical Genome Resource, 2021e | |
| *MEN1* | Multiple endocrine neoplasia type 1 | | ClinGen Clinical Genome Resource, 2021n | |
| *MLH1, MSH2, MSH6, PMS2* | Lynch syndrome | | ClinGen Clinical Genome Resource, 2021o | |
| *MUTYH* | *MUTYH*-associated polyposis | | ClinGen Clinical Genome Resource, 2021p | |
| *PTEN* | *PTEN* hamartoma tumor | | ClinGen Clinical Genome Resource, 2021s | |
| *RB1* | Hereditary retinoblastoma | | ClinGen Clinical Genome Resource, 2021ac | |
| *RET* | Multiple endocrine neoplasia type 2 | | ClinGen Clinical Genome Resource, 2021t | |
| *SDHAF2, SDHB, SDHC, SDHD* | Hereditary paragranglioma / pheochromocytoma | | ClinGen Clinical Genome Resource, 2021m | |
| *STK11* | Peutz-Jeghers syndrome | | ClinGen Clinical Genome Resource, 2021y | |
| *TSC1, TSC2* | Tuberous sclerosis | | ClinGen Clinical Genome Resource, 2021aa | |
| *TP53* | Li-Fraumeni syndrome | | ClinGen Clinical Genome Resource, 2021z | |
| *VHL* | Von Hippel Lindau syndrome | | ClinGen Clinical Genome Resource, 2021ab | |
|  |  | |  | |
| ***Cardiovascular Conditions*** | |  | |  |
| *ACTA2, MYH11* | | Non-syndromic aortopathies | | ClinGen Clinical Genome Resource, 2021a |
| *ACTC1, LMNA, MYBPC3, MYH7, MYL2, MYL3, PRKAG2, TNNT2, TNNI3, TPM1* | | Hypertrophic and dilated cardiomyopathies | | ClinGen Clinical Genome Resource, 2021b;g |
| *APOB, LDLR, PCSK9* | | Familial hypercholesterolemia | | ClinGen Clinical Genome Resource, 2021k;l |
| *COL3A1* | | Ehlers Danlos syndrome, vascular type | | ClinGen Clinical Genome Resource, 2021f |
| *DSC2, DSG2, DSP, PKP2, TMEM43* | | Arrhythmogenic right ventricular cardiomyopathy | | ClinGen Clinical Genome Resource, 2021r |
| *FBN1* | | Marfan syndrome | | ClinGen Clinical Genome Resource, 2021h |
| *GLA* | | Fabry disease | | ClinGen Clinical Genome Resource, 2021i |
| *KCNH2, KCNQ1, SCN5A* | | Long QT syndrome | | ClinGen Clinical Genome Resource, 2021j |
| *RYR2* | | Catecolaminergic polymorphic ventricular tachycardia | | ClinGen Clinical Genome Resource, 2021v |
| *TGFBR1, TGFBR2, SMAD3* | | Loeys-Dietz syndrome | | ClinGen Clinical Genome Resource, 2021w |
|  | |  | |  |
| ***Metabolic Conditions*** | |  | |  |
| *ATP7B* | | Wilson disease | | ClinGen Clinical Genome Resource, 2021d |
| *OTC* | | Ornithine transcarbamylase deficiency | | ClinGen Clinical Genome Resource, 2021q |
| *RYR1, CACNA1S* | | Malignant hyperthermia | | ClinGen Clinical Genome Resource, 2021u |

**REFERENCES, TABLE S2**

ClinGen Clinical Genome Resource (2021a). *Adult Summary Report, Gene/Gene Panel: ACTA2, FBN1, LOX, MYH11, PRKG1, SMAD3, TGFB2, TGFBR1, TGFBR2, Condition: Familial thoracic aortic aneurysms and dissections (FTAAD)* [Online]. Available: <https://actionability.clinicalgenome.org/ac/Adult/ui/stg2SummaryRpt?doc=AC134> [Accessed Feb 8 2021].

ClinGen Clinical Genome Resource (2021b). *Adult Summary Report, Gene/Gene Panel: ACTC1, CSRP3, MYBPC3, MYH7, MYL2, MYL3, PRKAG2, TNNI3, TNNT2, TPM1, Condition: Familial Hypertrophic Cardiomyopathy* [Online]. Available: <https://actionability.clinicalgenome.org/ac/Adult/ui/stg2SummaryRpt?doc=AC134> [Accessed Feb 8 2021].

ClinGen Clinical Genome Resource (2021c). *Adult Summary Report, Gene/Gene Panel: APC, Condition: Familial Adenomatous Polyposis* [Online]. Available: <https://actionability.clinicalgenome.org/ac/Adult/ui/stg2SummaryRpt?doc=AC048&version=23002> [Accessed Feb 8 2021].

ClinGen Clinical Genome Resource (2021d). *Adult Summary Report, Gene/Gene Panel: ATP7B, Condition: Wilson Disease* [Online]. Available: <https://actionability.clinicalgenome.org/ac/Adult/ui/stg2SummaryRpt?doc=AC028> [Accessed Feb 8 2021].

ClinGen Clinical Genome Resource (2021e). *Adult Summary Report, Gene/Gene Panel: BRCA1, BRCA2, Condition: Hereditary Breast and Ovarian Cancer* [Online]. Available: <https://actionability.clinicalgenome.org/ac/Adult/ui/stg2SummaryRpt?doc=AC133> [Accessed Feb 8 2021].

ClinGen Clinical Genome Resource (2021f). *Adult Summary Report, Gene/Gene Panel: COL3A1 Condition: Ehlers-Danlos Syndrome Type IV* [Online]. Available: <https://actionability.clinicalgenome.org/ac/Adult/ui/stg2SummaryRpt?doc=AC132> [Accessed Feb 8 2021].

ClinGen Clinical Genome Resource (2021g). *Adult Summary Report, Gene/Gene Panel: DMD, LMNA, TNNT2 Condition: Dilated cardiomyopathy* [Online]. Available: <https://actionability.clinicalgenome.org/ac/Adult/ui/stg2SummaryRpt?doc=AC134> [Accessed Feb 8 2021].

ClinGen Clinical Genome Resource (2021h). *Adult Summary Report, Gene/Gene Panel: FBN1, Condition: Marfan Syndrome* [Online]. Available: <https://actionability.clinicalgenome.org/ac/Adult/ui/stg2SummaryRpt?doc=AC100> [Accessed Feb 8 2021].

ClinGen Clinical Genome Resource (2021i). *Adult Summary Report, Gene/Gene Panel: GLA, Condition: Fabry Disease* [Online]. Available: <https://actionability.clinicalgenome.org/ac/Adult/ui/stg2SummaryRpt?doc=AC047> [Accessed Feb 8 2021].

ClinGen Clinical Genome Resource (2021j). *Adult Summary Report, Gene/Gene Panel: KCNQ1, KCNH2, SCN5A, Condition: Long QT Syndrome, types 1, 2, and 3* [Online]. Available: <https://actionability.clinicalgenome.org/ac/Adult/ui/stg2SummaryRpt?doc=AC131> [Accessed Feb 8 2021].

ClinGen Clinical Genome Resource (2021k). *Adult Summary Report, Gene/Gene Panel: LDLR, APOB, PCSK9 Condition: Heterozygous Familial Hypercholesterolemia* [Online]. Available: <https://actionability.clinicalgenome.org/ac/Adult/ui/stg2SummaryRpt?doc=AC057> [Accessed Feb 8 2021].

ClinGen Clinical Genome Resource (2021l). *Adult Summary Report, Gene/Gene Panel: LDLR, APOB, PCSK9 Condition: Homozygous Familial Hypercholesterolemia* [Online]. Available: <https://actionability.clinicalgenome.org/ac/Adult/ui/stg2SummaryRpt?doc=AC065> [Accessed Feb 8 2021].

ClinGen Clinical Genome Resource (2021m). *Adult Summary Report, Gene/Gene Panel: MAX, SDHA, SDHAF2, SDHB, SDHC, SDHD, TMEM127, Condition: Paragangliomas 1, 2, 3, 4, 5; Pheochromocytoma* [Online]. Available: <https://actionability.clinicalgenome.org/ac/Adult/ui/stg2SummaryRpt?doc=AC150> [Accessed Feb 8 2021].

ClinGen Clinical Genome Resource (2021n). *Adult Summary Report, Gene/Gene Panel: MEN1, Condition: Multiple Endocrine Neoplasia Type I* [Online]. Available: <https://actionability.clinicalgenome.org/ac/Adult/ui/stg2SummaryRpt?doc=AC077> [Accessed Feb 8 2021].

ClinGen Clinical Genome Resource (2021o). *Adult Summary Report, Gene/Gene Panel: MLH1, MSH2, MSH6, PMS2, EPCAM, Condition: Lynch Syndrome* [Online]. Available: <https://actionability.clinicalgenome.org/ac/Adult/ui/stg2SummaryRpt?doc=AC069> [Accessed Feb 8 2021].

ClinGen Clinical Genome Resource (2021p). *Adult Summary Report, Gene/Gene Panel: MUTYH, Condition: MUTYH-Associated Polyposis* [Online]. Available: <https://actionability.clinicalgenome.org/ac/Adult/ui/stg2SummaryRpt?doc=AC070> [Accessed Feb 8 2021].

ClinGen Clinical Genome Resource (2021q). *Adult Summary Report, Gene/Gene Panel: OTC, Condition: Ornithine Transcarbamylase Deficiency* [Online]. Available: <https://actionability.clinicalgenome.org/ac/Adult/ui/stg2SummaryRpt?doc=AC112> [Accessed Feb 8 2021].

ClinGen Clinical Genome Resource (2021r). *Adult Summary Report, Gene/Gene Panel: PKP2, DSP, DSC2, TMEM43, DSG2, JUP Condition: Arrhythmogenic Right Ventricular Dysplasia* [Online]. Available: <https://actionability.clinicalgenome.org/ac/Adult/ui/stg2SummaryRpt?doc=AC039> [Accessed Feb 8 2021].

ClinGen Clinical Genome Resource (2021s). *Adult Summary Report, Gene/Gene Panel: PTEN, Condition: PTEN Hamartoma Tumor Syndrome - Cowden Syndrome* [Online]. Available: <https://actionability.clinicalgenome.org/ac/Adult/ui/stg2SummaryRpt?doc=AC025> [Accessed Feb 8 2021].

ClinGen Clinical Genome Resource (2021t). *Adult Summary Report, Gene/Gene Panel: RET, Condition: Multiple Endocrine Neoplasia IIA, Familial Medullary Thyroid Cancer* [Online]. Available: <https://actionability.clinicalgenome.org/ac/Adult/ui/stg2SummaryRpt?doc=AC080> [Accessed Feb 8 2021].

ClinGen Clinical Genome Resource (2021u). *Adult Summary Report, Gene/Gene Panel: RYR1, CACNA1S, Condition: Malignant Hyperthermia Susceptibility* [Online]. Available: <https://actionability.clinicalgenome.org/ac/Adult/ui/stg2SummaryRpt?doc=AC076> [Accessed Feb 8 2021].

ClinGen Clinical Genome Resource (2021v). *Adult Summary Report, Gene/Gene Panel: RYR2, Condition: Catecholaminergic Polymorphic Ventricular Tachycardia* [Online]. Available: <https://actionability.clinicalgenome.org/ac/Adult/ui/stg2SummaryRpt?doc=AC042> [Accessed Feb 8 2021].

ClinGen Clinical Genome Resource (2021w). *Adult Summary Report, Gene/Gene Panel: SMAD3, TGFB2, TGFB3, TGFBR1, TGFBR2, Condition: Loeys-Dietz Syndrome* [Online]. Available: <https://actionability.clinicalgenome.org/ac/Adult/ui/stg2SummaryRpt?doc=AC067> [Accessed Feb 8 2021].

ClinGen Clinical Genome Resource (2021x). *Adult Summary Report, Gene/Gene Panel: SMAD4, BMPR1A, Condition: Juvenile polyposis syndrome* [Online]. Available: <https://actionability.clinicalgenome.org/ac/Adult/ui/stg2SummaryRpt?doc=AC066> [Accessed Feb 8 2021].

ClinGen Clinical Genome Resource (2021y). *Adult Summary Report, Gene/Gene Panel: STK11, Condition: Peutz-Jeghers Syndrome* [Online]. Available: <https://actionability.clinicalgenome.org/ac/Adult/ui/stg2SummaryRpt?doc=AC115> [Accessed Feb 8 2021].

ClinGen Clinical Genome Resource (2021z). *Adult Summary Report, Gene/Gene Panel: TP53, Condition: Li-Fraumeni Syndrome* [Online]. Available: <https://actionability.clinicalgenome.org/ac/Adult/ui/stg2SummaryRpt?doc=AC068> [Accessed Feb 8 2021].

ClinGen Clinical Genome Resource (2021aa). *Adult Summary Report, Gene/Gene Panel: TSC1, TSC2, Condition: Tuberous Sclerosis Complex (TSC)* [Online]. Available: <https://actionability.clinicalgenome.org/ac/Adult/ui/stg2SummaryRpt?doc=AC026> [Accessed Feb 8 2021].

ClinGen Clinical Genome Resource (2021ab). *Adult Summary Report, Gene/Gene Panel: VHL, Condition: Von Hippel-Lindau Syndrome* [Online]. Available: <https://actionability.clinicalgenome.org/ac/Adult/ui/stg2SummaryRpt?doc=AC027> [Accessed Feb 8 2021].

ClinGen Clinical Genome Resource (2021ac). *Pediatric Summary Report, Gene/Gene Panel: RB1, Condition: Retinoblastoma* [Online]. Available: <https://actionability.clinicalgenome.org/ac/Adult/ui/stg2SummaryRpt?doc=AC025> [Accessed Feb 8 2021].
