# Supplementary material for: Precision Population Medicine in Primary Care: The Sanford Chip Experience
Source: Front Genet. 2021 Mar 12;12:626845. doi: 10.3389/fgene.2021.626845 (PMC7994529; doi:10.3389/fgene.2021.626845)
Supplement: Supplementary Table 3 — Frequency of phenotypes/genotypes in pharmacogenomic genes. Results represent findings from the first 10,723 patients who enrolled in the Sanford Chip program. Counts vary by gene depending on the genes included on the Sanford Chip at the time patients received testing. Some genes from Supplementary Table 1 are omitted because they were only recently added to the Sanford Chip. [file Table_3.docx]

Table S3. Frequency of phenotypes/genotypes in pharmacogenomic genes. Results represent findings from the first 10,723 patients who enrolled in the Sanford Chip program. Counts vary by gene depending on the genes included on the Sanford Chip at the time patients received testing. Some genes from Table S1 are omitted because they were only recently added to the Sanford Chip.

| **Gene and phenotype or genotype** | **n (%)** |
| --- | --- |
| *CYP2C9* |  |
| Poor Metabolizer | 246 (2.3%) |
| Intermediate Metabolizer | 3,392 (31.6%) |
| Normal Metabolizer | 7,085 (66.1%) |
|  |  |
| *CYP2D6* |  |
| Poor Metabolizer | 691 (6.4%) |
| Intermediate Metabolizer | 4,069 (37.9%) |
| Normal Metabolizer | 5,582 (52.1%) |
| Ultra-rapid Metabolizer | 193 (1.8%) |
| Normal or Ultra-Rapid Metabolizer | 42 (0.4%) |
| Normal or Intermediate Metabolizer | 10 (0.1%) |
| Normal or Intermediate or Ultra-Rapid Metabolizer | 117 (1.1%) |
| Unknown Phenotype | 19 (0.2%) |
|  |  |
| *CYP2C19* |  |
| Poor Metabolizer | 293 (2.7%) |
| Intermediate Metabolizer | 2,818 (26.3%) |
| Normal Metabolizer | 4,225 (39.4%) |
| Rapid Metabolizer | 2,880 (26.9%) |
| Ultra-rapid Metabolizer | 507 (4.7%) |
|  |  |
| *CYP2C9/VKORC1* |  |
| Low Warfarin Sensitivity | 6,141 (63.7%) |
| Intermediate Warfarin Sensitivity | 3,174 (32.9%) |
| Normal Metabolizer | 1 (0.0%) |
| High Warfarin Sensitivity | 325 (3.4%) |
|  |  |
| *VKORC1* |  |
| Low Warfarin Sensitivity | 4,177 (39.0%) |
| Intermediate Warfarin Sensitivity | 4,957 (46.2%) |
| High Warfarin Sensitivity | 1,589 (14.8%) |
|  |  |

| *CYP4F2* |  |
| --- | --- |
| *CYP4F2*1* | 1,344 (53.1%) |
| *CYP4F2*3* | 1,187 (46.9%) |
|  |  |
| *CYP2C* gene cluster |  |
| Reference | 1,754 (69.3%) |
| rs12777823 | 777 (30.7%) |
|  |  |
| *DPYD* |  |
| Poor Metabolizer | 2 (0.0%) |
| Intermediate Metabolizer | 216 (2.0%) |
| Normal Metabolizer | 1,505 (98.0%) |
|  |  |
| *TPMT* |  |
| Poor Metabolizer | 29 (0.3%) |
| Intermediate Metabolizer | 135 (1.3%) |
| Intermediate or Poor Metabolizer | 789 (7.4%) |
| Normal Metabolizer | 9,767 (91.1%) |
|  |  |
| *SLCO1B1* |  |
| Poor Function | 275 (2.6%) |
| Decreased Function | 2,804 (26.1%) |
| Normal Function | 7,644 (71.3%) |
|  |  |
| *CYP3A5* |  |
| Poor Metabolizer | 9,124 (85.1%) |
| Intermediate Metabolizer | 1,502 (14.0%) |
| Normal Metabolizer | 97 (0.9%) |
|  |  |
| *IFNL3* |  |
| Favorable response | 1,096 (43.3%) |
| Unfavorable response | 1,435 (56.7%) |
